# Determination of Selenium Species in Muscle, Heart, and Liver Tissues of Lambs Using Mass Spectrometry Methods

**DOI:** 10.3390/ani10050808

**Published:** 2020-05-07

**Authors:** Andrzej Gawor, Anna Ruszczynska, Marian Czauderna, Ewa Bulska

**Affiliations:** 1 Biological and Chemical Research Centre, Faculty of Chemistry, University of Warsaw, Zwirki i Wigury 101, 02-089 Warsaw, Poland; agawor@chem.uw.edu.pl (A.G.); aruszcz@chem.uw.edu.pl (A.R.); 2 The Kielanowski Institute of Animal Physiology and Nutrition, Polish Academy of Sciences, Instytucka 3, 05-110 Jablonna, Poland; m.czauderna@ifzz.pl

**Keywords:** selenium supplementation, selenium species, mass spectrometry

## Abstract

**Simple Summary:**

The objective of the project was to evaluate speciation of selenium (Se) in various tissues (the liver, heart, or muscles) obtained from lambs fed with a diet enriched with an inorganic and organic chemical form of Se (i.e., selenite and Se-enriched yeast, respectively).

**Abstract:**

Identification and quantification of the selenium species in biological tissues is imperative, considering the need to properly understand its metabolism and its importance in various field of sciences, especially nutrition science. Although a number of studies deals with the speciation of selenium, speciation analysis is still far from being a routine task, and so far strongly depends on the type of the samples. We present a study aimed to examine speciation analysis of Se in tissues of livers, muscles, and hearts obtained from lambs, namely in liver, muscle, and heart. The studied lambs were fed with the diet enriched with an inorganic (as sodium selenate) and organic chemical form of Se (as Se-enriched yeast) compounds with simultaneous addition of fish oil (FO) and carnosic acid (CA). The first part of the work was focused on the optimization of the extraction procedure of selenium compounds from tissues. Next, hyphenated high performance liquid chromatography and inductively coupled plasma mass spectrometry (HPLC–ICP–MS) was used for the identification of five seleno-compounds—Se-methionine (SeMet), Se-cystine (SeCys2), Se-methyl-Se-cysteine (SeMetSeCys), and Se(IV) and Se(VI). Verification of the identified seleno-compounds was achieved using triple-quadrupole mass spectrometer coupled to high performance liquid chromatography (HPLC–ESI–MS/MS). The applied procedure allowed for quantitative analysis of SeMet, SeCys2, and SeMetSeCys, in biological tissues. The developed analytical protocol is feasible for speciation analysis of small molecular seleno-compounds in animals samples.

## 1. Introduction

Interest in selenium speciation in biological materials has been increasing since selenium (Se) (rarely considered a metalloid) was recognized as a crucial trace element in living systems. Selenium is an essential component mandatory for the biosynthesis of 25 selenoproteins (including thioredoxin reductase, glutathione peroxidase, and iodothyronine deiodinases). Selenium compounds have antioxidant functions [1], have anticancer properties [2,3,4], affect the cardiovascular system [5], and reduce the activity of viruses including HIV [6]. A proper diet or supplementation with selenium compounds allows to maintain a proper level of this element in the body. The selenium supplements could be either in the form of inorganic mineral salts, such as sodium selenate (Na_2_SeO_4_) or selenite (Na_2_SeO_3_), or in the organic form of Se-enriched yeast. The last one contains a mixture of organic seleno-compounds with Se-methionine (SeMet) being the predominant source of Se. Dietary SeMet and Se-cysteine (SeCys) are naturally occurring amino acids that have structures similar to methionine and cysteine, except that Se replaces sulfur [7].

Recent studies documented that carnosic acid (CA), a phenolic diterpene, reveals strong antimicrobial, anticarcinogenic, and antioxidative properties [8,9,10]. Moreover, CA efficiently protects lipids from oxidative damages, as CA (similar to tocopherols or Se-compounds) scavenges reactive oxygen species (ROS). During this process, dietary CA especially prevents oxidation of highly unsaturated fatty acids (HUFA) in mammal tissues [9,10]. Previous investigations indicated that CA added to diets affects a rumen microorganism profile, a microbiota activity, the yield of bacterial isomerization and biohydrogenation of unsaturated fatty acids (UFA) and, hence, results in the formation of rumen volatile fatty acids and the composition of fatty acids (FA), in the ruminants’ body [9,10]. Similarly, dietary fish oil (FO) rich in HUFA, is able to change ruminal biohydrogenation by reducing the enzymatic isomerization capacity of UFA in the rumen of lambs or cows [11]. Indeed, dietary long chain polyunsaturated fatty acids (LPUFA), especially the very long chain highly unsaturated fatty acids (VLC-HUFA), decreased the activity (e.g., isomerase activity) and growth of microbiota in the rumen. As a consequence, the percentage of detrimental saturated FA in ruminants’ tissues decreases, whereas the percentage of UFA (especially pro-health n-3VLC-HUFA) in tissues, increases [11]. Most importantly, especially n-3VLC-HUFA derived from FO have many important physiological effects on a range of cellular functions that might reduce the onset of heart diseases and decrease mortality among patients with coronary heart disease. Moreover, FO rich in n-3VLC-HUFA improve brain function. Supplementation of FO (rich in pro-health n-3VLC-HUFA) and antioxidants (like Se-compounds and CA) to diets of farm animals is an effective way to improve the nutritional quality of animal products for humans. In fact, increasing the share of FA to VLC-HUFA in diets of farm animals requires effective protection against oxidative damages. Therefore, special attention should be given to addition of antioxidants (like seleno-compounds or CA) to animal diet enriched in FO (rich in VLC-HUFA) [1,2,3,4,5]. Indeed, the adequate supply of oils (rich in VLC-HUFA) and antioxidants in the diet ensure balance in the animals’ organism and reduces the oxidative stress and oxidative degradation of pro-health n-3VLC-HUFA in the edible part of the carcass of farm animals. Taking into account the above-mentioned facts, we hypothesized that odorless FO (rich in VLC-HUFA), CA, and Se-compounds (like selenate or Se-enriched yeast) added to the diet would modify profiles of physiologically important Se-compounds in lamb tissues. Moreover, it is expected that the chemical form of dietary Se-compounds has a significant impact on the profile of selenium species in analysed tissues. Indeed, the physiological properties, chemical activity and bioaccumulation of Se depended upon a chemical form of selenium in mammal tissues. Therefore, the identification and quantification of selenium species in biological tissues is crucial for understanding its metabolism and importance in biology, clinical chemistry, toxicology, and nutrition science.

Due to the usually low concentration of Se in biological tissues as well as its complex matrix, it is necessary to apply selective and sensitive analytical techniques for its determination. Hydride generation atomic absorption spectrometry (HG–AAS) [12,13], electrothermal atomic absorption spectrometry (ET–AAS) [14,15], radiochemical and instrumental neutron activation analysis (RNAA and INAA) [16], and inductively coupled plasma mass spectrometry (ICP–MS) [17,18] are frequently used for this purpose. In order to perform speciation, it is necessary to use separate techniques coupled to sensitive detection methods, i.e., elemental or molecular mass spectrometry [19,20,21,22,23,24,25,26,27,28,29,30]. Among the mentioned techniques ICP–MS is the most powerful element-specific method for total Se determination in biological samples. This type of analysis is carried out on samples after their digestion in low pH, using nitric acid (HNO_3_), with frequent addition of hydrogen peroxide (H_2_O_2_), i.e., especially for biological materials rich in fat. Recently, coupling an high performance liquid chromatography (HPLC) to ICP–MS has become the most popular approach in speciation analysis of Se [31].

According to the International Union of Pure and Applied Chemistry (IUPAC) statement [32], selection of a suitable extraction method for individual compounds is one of the most difficult tasks in speciation analysis, mainly due to their very low concentration and presence in complex matrices. The extraction procedures of selenium species from biological samples involve boiling in water [33,34,35], use of Tris-HCl buffer, acid hydrolysis [36] and enzymes such as protease [36], pronase [37], proteinase K [38], and protease-lipase [20]. Extraction is commonly supported by sonication or mechanical shaking of the sample with added inorganic and organic solvents.

Increment of the Se content and its species in animal tissues depends, in particular, on the quantity and form of Se to which the animal is exposed. Selenium supplementation of livestock has become routine in agriculture. This is due to its role in the prevention of a variety of economically significant diseases in farm animals. Speciation of selenium has been widely studied in biological tissues and fluids, in animals such as rats [39], sheep [20,40,41,42,43], chickens [40,42], turkeys [42], ducks [42], pigs [42,44,45], and marine organisms [24,44,46,47], but still very little practical information is available on either the persistency of Se or the distribution of selenium-containing amino acids.

The objective of the project was speciation analysis of Se in various tissues (e.g., the liver, heart, or muscles) obtained from examined sheep fed with the feed enriched with FO, CA, an inorganic selenium compound (as sodium selenate; Se(VI)) or organoselenium compounds (as Se-enriched yeast; SeY). The supplements added to the lambs’ diets did not negatively affect the welfare and general health status of the examined animals, since no pathological changes and macroscopic lesions were observed in the lamb [17,41,43]. In fact, diets containing up to 2 mg/kg Se are not toxic for mammals (ruminants in particular) [48,49,50].

## 2. Materials and Methods

### 2.1. Chemicals and Reagents

Analytical reagent grade chemicals were purchased from Sigma Aldrich (Darmstadt, Germany), EMD Millipore (Darmstadt, Germany), LGC Standards (Teddington, UK), and Baker (Deventer, Netherlands). The following selenium standards were used—seleno-DL-methionine (Sigma Aldrich, Germany), seleno-L-cystine (Sigma Aldrich, Germany), Se-methyl-seleno-L-cysteine (Sigma Aldrich, Germany), Se(IV) standard solution (LGC Standards, UK), and Se(VI) standard solution (LGC Standards, UK). Series of dilutions of ICP multi-element standard Merck VI (Merck, Darmstadt, Germany) was used for stock solutions preparation. Certified Reference Materials (CRM) were used—bovine liver tissue NIST 1577c (NIST, Gaithersburg, MD, USA), MODAS-4 cormorant tissue (Consortium MODAS, Warsaw, Poland) and SELM-1 (National Research Council, Ottawa, ON, Canada). Analytical grade nitric acid, 65%, (EMD Millipore, Darmstadt, Germany) was used for sample digestion. Samples and standards were diluted with deionized water obtained by the Milli-Q System (18.2 MΩ cm; EMD Millipore, Darmstadt, Germany). Formic acid (∼98%) used for liquid chromatographic purposes was purchased from Honeywell Chemicals (Bracknell, UK).

Carnosic acid (CA) was supplied by Hunan Geneham Biomedical Technology Ltd. (The People’s Republic of China; Changsha Road). Odorless fish oil (FO), rich in n-3LPUFA) and rapeseed oil (RO) were purchased from AGSOL company (Pacanow, Poland). The energy content of odorless FO and RO was 36.8 MJ/kg oil and 37.0 MJ/kg oil, respectively [17]. The mineral and vitamin mixture (ID number of premix: aPL-1405002p) was supplied by POLFAMIX OK Trouw Nutrition (Grodzisk Mazowiecki, Poland). Se-enriched yeast (SeY; *Se-Saccharomyces cerevisiae* enriched in organoselenium compounds) was purchased from Sel-Plex^®^ (Alltech In., Lexington, KY, USA).

### 2.2. Samples

The objects of the study were muscle, liver, and heart tissues of lambs on an experimental diet with Se supplementation. All studies on corriedale male lambs were conducted under the authority of the III^rd^ Local Commission of Animal Experiment Ethics at the Warsaw University of Life Sciences (Poland); animal handling procedures and welfare guidelines were strictly followed throughout the entire period of studies carried out on lambs. Breeding of animal and tissue collection were carried out at the Kielanowski Institute of Animal Physiology and Nutrition Polish Academy of Sciences (Jablonna, Poland). Thirty lambs in the age range of 82 days to 90 days and body weight of 24.3 kg ± 1.6 kg at the beginning of experiments, were individually penned as described in our previous papers [17,43,49]. During a 3-week preliminary period, the lambs had ad libitum access to fresh drinking water and a basal diet (BD) with mineral and vitamin premix [17,43], enriched in 3% or 2% rapeseed oil (RO) and 1% odorless FO (Table 1). The BD (the standard concentrate-hay diet [17,51]) consists of the following components [17,43]—a mixture of soybean meal and barley meal, wheat starch, meadow hay, and mineral–vitamin premix. The chemical profiles of the ingredients in the BD as well as the fatty acid concentrations in odorless FO and RO were presented in our recent papers [17,43] or in the supplementary materials associated with this paper.

After the 3-week adaptation period, the lambs were divided into 5 groups of 6 animals (Table 1). Then, the animals were fed for 35 days on the BD containing 3% RO (the control diet—the RO diet), the BD including 1% FO and 2% RO (the FO diet), the BD enriched in 2% RO, 1% FO, and 0.1% CA (the CA diet), the BD enriched in 2% RO, 1% FO, 0.1% CA, and SeY (the CASeY diet), and the BD enriched in 2% RO, 1% FO, 0.1% CA, and Se(VI) (the CASeVI diet) (Table 1). The RO diet and all supplemented feeds were prepared daily and given to lambs twice a day (7.30 a.m. and 4.00 p.m.), in equal portions. All lambs were fed the same amount of freshly prepared diets with appropriate additives (Table 1). All lambs were separately given the RO diet and four supplemented diets as meadow hay and concentrate. The portions of diets were weekly adjusted to both, the body weight of lambs and their nutritional requirements, according to Strzetelski et al. [51]. The offered doses were wholly consumed by the lambs. The average daily diet intake per lamb was 1.08 kg. The control diet and all experimental diets were formulated to be isoenergetic and isoproteinous. Water for the animals was available ad libitum.

At the end of the 35-day experimental period, after 12-hour fasting, all lambs were deprived of consciousness by intramuscular injections of xylazine (about 0.4 mg/kg of body mass) and then slaughtered. The lambs’ deprivation of consciousness and tissue collection was carried out in accordance with the Regulation of the Council of the European Union (EC) No. 130 1099/2009 of 24 September 2009 on the Protection of Animals at the Time of Killing and under the supervision of the III^rd^ Local Ethics Committee on Animal Experiments at the Warsaw University of Life Sciences – SGGW (Warsaw, Poland). Immediately after the slaughter, the heart, liver, and muscles (longissimus dorsi muscle) were removed from each lamb. The collected hearts, livers, and muscles were homogenized and then stored in sealed tubes at −32 °C, until analytical investigation.

### 2.3. Instrumentation

#### 2.3.1. Sample Preparation, ICP–MS

The microwave system Ultra Wave (Milestone, Sorisole, Italy) with closed vessels was used for digestion of samples and their extracts. The extracts solutions were centrifuged using 5804/5804R centrifuge (Eppendorf, Enfield, CT, USA) and subsequently the supernatants were concentrated in the vacuum centrifuge (Eppendorf, USA). Isotope specific detection was achieved using quadrupole mass spectrometer with inductively coupled plasma ionization, ICP–MS, (Nexion 300D, Perkin Elmer, Boston, MA, USA) with a liquid sample introduction system consisting of Meinhard nebulizer and quartz cyclonic spray chamber. Spectrometer parameters were optimized daily in order to obtain the maximal sensitivity and stability. The ICP–MS conditions were as follows—a radio frequency power RF 1300 W, flow rate of plasma gas 15 L/min auxiliary gas 1.2 L/min, and nebulizer gas 0.8 L/min, dwell time 100 ms. The isotope ^82^Se with 8.7% of natural abundance was used for quantification, due to a large effect of polyatomic interferences (^40^Ar^40^Ar) on isotope ^80^Se.

#### 2.3.2. HPLC–ICP MS

Agilent1260 Infinity high performance liquid chromatography (Agilent Technologies, Santa Clara, CA, USA) with anion exchange Hamilton PRP-X100 (250 mm × 4.6 mm, particle size 10 µm) column (Hamilton, Reno, NV, USA) coupled with polyetheretherketone (PEEK) tubing to Elan 6100 DRC ICP–MS system (Perkin Elmer Sciex, QC, Canada), was used for tissue extract analysis.

#### 2.3.3. HPLC–ESI–MS/MS

High performance liquid chromatography coupled with tandem mass spectrometry were performed using a Nexera X2 LC-30AD UHPLC system coupled to a triple quadrupole MS-8060 mass spectrometer (both Shimadzu, Japan) equipped with an electrospray ionization source (ESI) and Hypercarb Porous Graphitic Carbon (PGC) Hypercarb column (150 mm × 4.6 mm, particle size 5 μm) purchased from Thermo Scientific (Bartlesville, OK, USA).

### 2.4. Experiment

#### 2.4.1. Freeze-Drying of Tissues

In order to ensure stability of biological material during storage, freeze-drying (lyophilization) of the collected tissues (temperature: −15 °C, pressure: 1 mbar, time: 12 h) was carried out. Lyophilization allows for both the structure and the chemical composition of the sample to remain intact. This procedure has long been used in laboratories to preserve a wide range of materials sensitive to external factors, such as tissues, plasma, proteins, pharmaceuticals, or microorganisms. The samples were homogenized by mortar and pestle and stored at 4 °C before conducting proper sample preparation.

#### 2.4.2. Extraction Procedure

The efficiency of the extraction process depends primarily on the type of solvent and the conditions of the process being carried out. The following solutions were investigated for their eventual use in extracting selenium species from lamb tissue—(i) 0.5% protease/lipase in water, (ii) 1% of sodium dodecyl sulfate in water, and (iii) water for their eventual use in extracting from selenium species from lamb tissue. The selection of the appropriate extraction mixture and the optimization of the extraction process were carried out using mass to charge ratio (m/z) monitoring for selenium isotopes (^82^Se and ^78^Se) through ICP–MS. The samples in the amount of 0.1 g were extracted with 5 mL extraction media. Extraction was conducted in 37 °C over 21 h, supported by magnetic stirring. The extraction solutions were centrifuged for 20 min at 10,000× *g*, supernatants were filtered through 0.45-μm membrane filters. Extraction yield determination was performed after dilution of extracts in 1% aqueous nitric acid. The extracts were kept at a temperature of −15 °C, before the chromatographic separation was carried out.

#### 2.4.3. Total Se Determination

Digestion of 1 mL of sample extracts in nitric acid was performed under the following programme—20 min up to 220 °C and 15 min at 220 °C. Cooled digests were diluted with water and analyzed by ICP–MS. Five point calibration curve with correlation coefficient R^2^ = 0.9999 was achieved in the range from 0.5 µg/L to 50 µg/L and the procedure was validated by the analysis of reference materials (Modas-4 and NIST 1577c).

#### 2.4.4. HPLC–ICP–MS Analysis

The first stage of the research was to optimize the separation conditions of selenium compounds using HPLC–ICP–MS. Based on previous report [21], the Hamilton PRP-X100 anion exchange column (4.6 mm × 150 mm, particle size 5 μm) was chosen to separate the selenium compounds. Ammonium acetate with a concentration of 5 mmol/L (solvent A), 150 mmol/L ammonium acetate (solvent B), and pH = 4.7 were used as mobile phase. Mobile phase flow rate of 1 mL/min, injection volume of 100 μL were used. A mixture of available standards of selenium compounds, such as—Se-cystine (SeCys2), Se-methyl-Se-cysteine (SeMetSeCys), Se-methionine (SeMet) as well as selenate(IV) and selenate(VI), containing 10 μg/L of Se was used to optimize the separation process. By using a gradient elution, a good separation was achieved within 25 min. The measurements were performed for ^78^Se isotope. The limits of detection (LOD) for test compounds containing selenium were calculated as the average signal plus three times the standard deviation of the signal for the blank sample and were 36 μg/kg for SeMet, 48 μg/kg for SeCys2, 39 μg/kg for SeMetSeCys, 4 μg/kg for Se(IV), and 6 μg/kg for Se(IV).

Tissue extracts were concentrated in a vacuum centrifuge to dryness and re-suspended in 1% aqueous nitric acid to a final volume of 1 mL. In order to identify Se-compounds in tissue extracts, the retention times of the recorded signals were compared with those for the available Se-standards.

#### 2.4.5. LC–ESI–MS/MS Analysis

Studies with LC–ESI–MS/MS were performed using a Nexera X2 LC-30AD UHPLC system coupled to a triple quadrupole MS-8060 mass spectrometer (both from Shimadzu, Japan) equipped with an electrospray ionization source (ESI). The analysis was carried out in the MRM (Multiple Reaction Monitoring) follow-up mode, for three compounds—SeMet, SeCys2, and SeMetSeCys. All analytes were detected in the positive ionization mode. The mass spectrometry parameters were optimized using the standards (10 μg/L) of the individual compounds via auto-optimization with direct injection. The ESI–MS conditions were as follows—interface voltage: 3.0 kV; desolvation line temperature: 250 °C; nebulizer gas N_2_: 3.0 L/min; heating gas N_2_: 10 L/min; heat block temperature: 400 °C; interface temperature: 300 °C, and drying gas N_2_: 10 L/min. Argon was used as collision gas. In the MRM mode, two m/z transitions with the optimal selectivity and highest sensitivity were selected to confirm each analyte. The most intense transition was used for quantification and the less intense ion signals were used for qualitative analysis. The optimized MS/MS parameters are shown in Table 2.

The conditions for chromatographic separation were developed using Porous Graphitic Carbon (PGC) Hypercarb column (150 mm × 4.6 mm, particle size 5 μm) purchased from Thermo Scientific (USA). The mobile phase consisted of 0.1% of formic acid in water (solvent A) and 0.1% of formic acid in acetonitrile (solvent B) with increasing organic phase gradient; the mobile phase flow was 0.5 mL/min, the temperature of the column was 40 °C, and the injection volume was 10 μL. The initial mobile phase composition was 3% B, increasing to 95% B over 12 min, then held constant at 95% B for 5 min, and was finally brought back to the initial condition of 3% B for re-equilibration of chromatographic conditions. Extracts from the tissue were concentrated in a vacuum centrifuge to dryness and re-suspended in 0.1 % aqueous formic acid in a final volume of 100 μL. Data acquisition and integration of chromatographic area peaks corresponding to selenium-containing compounds used for quantitative analysis was performed using the LabSolutions software (software version 5.96, Shimadzu, Kyoto, Japan). The procedure used was validated. Certified reference materials for speciation analysis of Se in animal samples are not currently available. In order to calculate recovery factor and evaluate the accuracy of SeMet determinations, the certified reference material selenised yeast SELM-1 was used. The reference solutions and the examined tissue extracts were prepared in triplicates. The correlation coefficient (R^2^) was greater than 0.998 for each calibration curve. The limits of detection (LOD) for test compounds containing selenium were calculated as the average signal plus three times the standard deviation of the signal for the blank sample and were 26 μg/kg for SeMet, 32 μg/kg for SeCys2, and 25 μg/kg for SeMetSeCys.

## 3. Results

The total Se concentrations in dry tissues from liver, heart, and muscles, after selenium supplementation are given in Table 3. The analysis of samples from group I, group II, and group III gave the lowest selenium levels (243.7–257.1 μg/kg) for muscle samples, was similar for heart, and gave the highest levels for liver (700.1–853.5 μg/kg).

The selection of sample preparation procedure for selenium speciation analysis is going to be dependent on the matrix, the chemical form of selenium expected in the sample, and the instrumentation selected for further separation and identification of the species [31]. Following extraction media were investigated—(i) 0.5% protease/lipase in water, (ii) 1% of sodium dodecyl sulfate in water, and (iii) water. The determination of the total Se content in tissue extracts was performed in order to select the extracts with highest Se concentration thus increasing the probability of identification of low abundant Se-compounds during speciation analysis. The extraction efficiencies for (i) 0.5% protease/lipase in water, (ii) 1% of sodium dodecyl sulfate in water, and (iii) water were 85%, 76%, and 55%, respectively.

**Figure 1:**
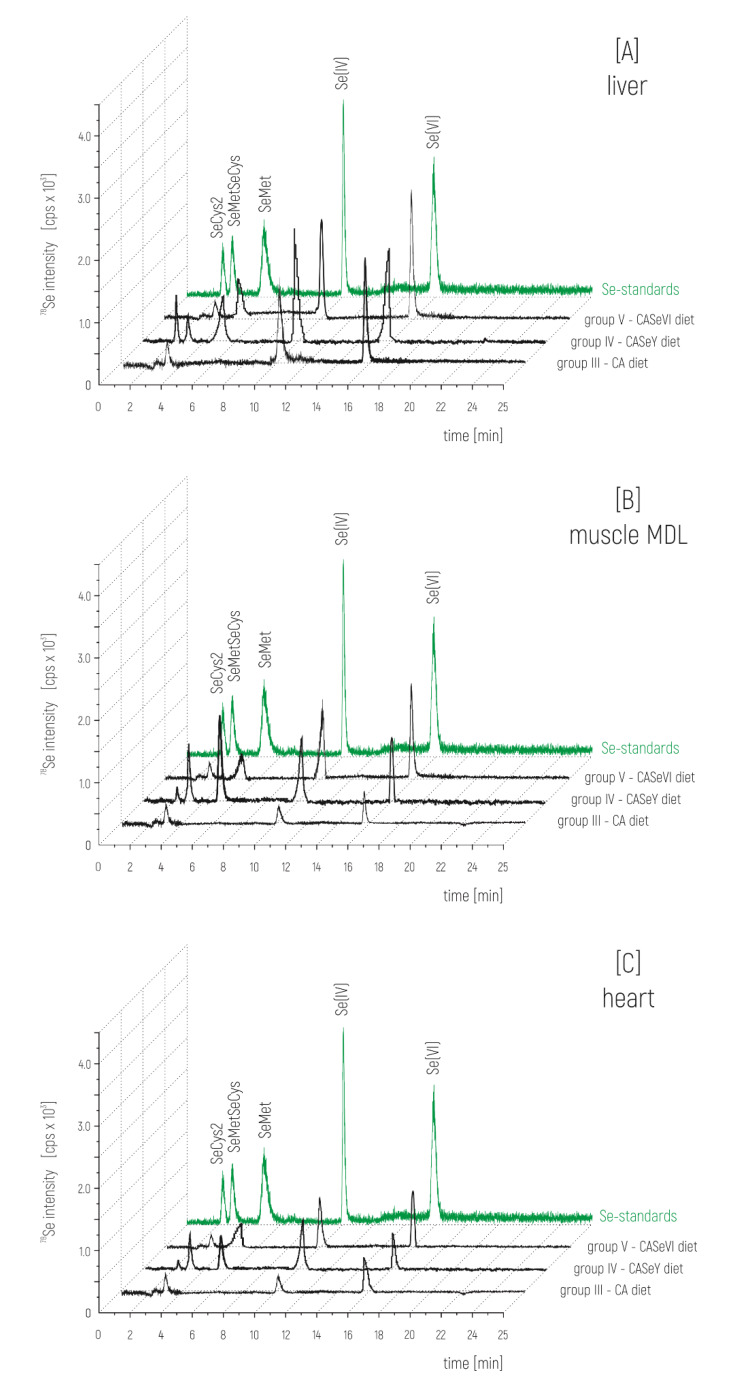
Chromatograms of ^78^Se signal obtained using HPLC–ICP–MS for analysis of (**A**) liver, (**B**) muscle MDL (longissimus dorsi muscle), and (**C**) heart from lambs, after supplementation with Se-compounds.

In order to evaluate selenium speciation in animals fed with a selenium-enriched diet, the complementary investigation towards identification of chemical compounds of Se was performed. The contents of various chemical selenium species in the liver, muscle, and heart tissues were determined by the anion-exchange HPLC followed by ICP–MS detection of ^78^Se isotope. Based on the results of extraction efficiencies, 0.5% protease/lipase in water solution was used for extraction of selenium species. The applied chromatographic conditions allowed the obtainment of sharp peaks and demonstrated a good separation efficiency within 25 min. The registered retention times for the standards were for SeCys2—2.10 min; SeMetSeCys—2.88 min; SeMet—4.95 min; Se(IV)—9.91 min, and Se(VI)—15.25 min (Figure 1). Matching the retention times of commercially available standards with the peaks in animal tissue extracts indicates the presence of Se(IV), Se(VI), and SeMetSeCys in all of them, and SeMet in supplemented groups IV and V. Peak corresponding to the presence of SeCys2 occurred only in samples from group IV supplementation. The identification of the obtained signals on the bases of comparison with commercially available standards needed to be confirmed through further identification.

In order to confirm the presence of the Se-compounds identified by HPLC–ICP–MS, high performance liquid chromatography coupled with tandem mass spectrometry equipped with an electrospray ionization source (ESI) was applied. Extracts of selected tissues were concentrated in a vacuum centrifuge and subjected to HPLC–ESI–MS/MS analysis. The retention time for SeMet, SeCys2, and SeMetSeCys were 5.82 min, 5.06 min, and 4.96 min, respectively. As a result of the HPLC–ESI–MS/MS analyses the identity of all selenium metabolites, previously identified by HPLC–ICP–MS was confirmed and quantified. The results of quantitative analysis of the selected seleno-compounds in liver, longissimus dorsi muscle (MLD) and heart are shown in Table 3.

## 4. Discussion

As expected, no adverse symptoms (like vomiting or diarrhea) in lambs fed with the control diet and all experimental diets enriched in FO, CA, SeY, or Se(VI) were observed. Feeding animals with the CASeVI diet increased (*p* ≤ 0.05) BWG and BW of lambs and worsened FCE, when compared to lambs fed with the control, CA and CASeY diets (Table 1). The control and all experimental diets had almost identical chemical composition, and differed only in dietary supplements. Therefore, the differences in the profile of seleno-compounds in the assayed tissues resulted from supplements added to the lambs’ diet.

The samples after supplementation of organic and inorganic forms of Se (IV and V group) showed a clear increase in Se content, in comparison to control diet (I, II, and III group), especially in liver (1.8-fold). The increases in muscle (1.2-fold) and heart (1.4-fold) were less noticeable. The obtained value of Se content in animal tissue after organic and inorganic Se-supplementations showed that organic forms were absorbed more efficiently than inorganic ones, which was in close agreement with the reported values of several studies [40,48,49]. Moreover, our current research as well previous studies [41,52,53] documented that the liver stored more selenium in comparison to other internal organs or tissues of animals fed with Se-compound-supplemented diets. In fact, the liver accumulated high levels of dietary Se-compounds because the liver is the first internal organ encountered by selenium species after Se-compound absorption in the intestine [54]. The trans-sulfuration pathway is more active in the liver than in other mammal tissues. Therefore, the liver is the major internal organ in which Se as SeMet is efficiently metabolized. Similarly, in the liver, selenide or catabolites of organoselenium compounds enter the pathway of Se-protein biosynthesis.

Depending on the tissue examined, 3 to 5 different forms of Se were identified by HPLC–ICP–MS (Figure 1). The difference in the intensity of signals corresponding to individual Se compounds suggests the effect of diet on changing their profile. This suggestion was confirmed by quantitative analysis by HPLC–ESI–MS/MS. The content of such compounds like SeMet, SeCys2, and SeMetSeCys (Table 3) shows the change in the profile of Se-compounds, depending on the type of diet and supplemented compound, organic or inorganic. The addition of SeY and other animal feed additives has an impact on the higher content of identified selenium organic forms such as SeCys, SeMetSeCys, or SeMet compared to other diets. The positive effect of other animal feed additives (FO, CA) on the change in the profile of Se-compounds in the examined animal tissues is also observed. Selenium in its various chemical forms appears to be well-absorbed from the diet. Organic Se-compounds (like SeCys, SeCys2, and especially SeMet) are deposited into the animal tissues more efficiently than dietary inorganic Se-compounds (like Se(IV) or Se(VI)) [40,48,49]. In foods of animal origin, supplementation with organic Se-compounds compared with inorganic Se-compounds results in meat of higher Se concentration, which was also confirmed by the results presented in Table 3. Beneficial effects of organic Se in dairy lamb nutrition can be associated with improvements to health and production of the animals, as a consequence of improved Se status. Selenium, in a form of SeMet, is incorporated into body proteins, which enables Se storage. This process takes place in organs and cells, which are characterized by high rates of protein biosynthesis, such as skeletal muscles, pancreas, erythrocytes, liver, kidneys, stomach, and the gastrointestinal mucosa [55]. Functional Se body pool can also be increased by the conversion of SeMet to SeCys [50]. Degradation of SeMet-containing proteins, which takes place in the liver, results in the production of methylated selenium compounds. Those are excreted predominantly in the urine, regulating selenium metabolism. Organic seleno-compounds are better absorbed and retained in comparison to inorganic seleno-compounds, i.e., selenite [17,41]. Results of various studies [17] that included orally supplementing lambs with Se, showed that Se accumulation in liver and heart was greater than that in the case of whole blood or MLD muscle.

## 5. Conclusions

The most important scientific novelty of our current studies was analyzing the effect of simultaneous addition of CA, FO (rich in pro-health n-3VLC-HUFA), CA, and Se (SeY in particular), to feed on the profile of physiologically important seleno-compounds in lamb tissues.

The use of an HPLC–ICP–MS and HPLC–ESI–MS/MS allowed for the speciation of seleno-metabolites in extracts of different animal tissues—liver, MLD muscle, and heart. Verification of identified seleno-compounds by HPLC–ICP–MS was achieved using HPLC–ESI–MS/MS. The obtained results of quantitative analysis of SeMet, SeCys2, and SeMetSeCys are important for the understanding of the metabolism of Se in animal tissues and to those who have an interest in animals.

The presented results of the work proved that it is possible to use diets enriched in CA, FO, and seleno-compounds, both in animal feed to improve the growth performance parameters (like FCE, BW, or BWG) of farm animals and the quality of meat intended for human consumption. The use of FO (rich in n-3VLC-HUFA), CA, and selenium-containing animal feed additives can be an excellent ways to produce functional foods that can meet the dietary needs of Se, in particular in areas that are low in this element and contribute to the prevention of various health conditions.

In conclusion, we argued that the current original investigations provide very important guidance for scientists carrying out new research to improve animal performance, health, and welfare, as well as the nutritional quality of animal products (especially rich in pro-health organic seleno-compounds, n-3VLC-HUFA, CA, and its metabolites), and thus, human diets.

## Figures and Tables

**Table 1:** The scheme of experiments carried out on lambs, additives added to the rapeseed oil (the RO diet, the control diet) and four experimental diets: fish oil diet (the FO diet), carnosic acid diet (the CA diet), carnosic acid selenium yeast diet (the CASeY diet) and carnosic acid selenium(VI) (the CASeVI diet), feed conversion efficiency (FCE), body weight (BW), and body weight gain (BWG) of sheep [43].

Group	Supplements Added to the Basal Diet (BD)	FCE for 35 Days of the Experimental Period (kg BWG/kg Diet Intake)	The Body Weight of Lambs		BWG ^3^ (kg)
			BW_initial_^1^ (kg)	BW_35days_^2^ (kg)	
**Group I ^4^** **(control group)**	3% RO (the RO diet ^6^)	0.150 ± 0.001 ^a,α^	30.7 ± 2.5 ^a^	36.3 ± 2.8 ^a^	5.6 ± 1.0 ^a,α^ (0.16 ± 0.03)
**Group II ^5^**	2% RO and 1% FO (the FO diet ^6^)	0.189 ± 0.001 ^a,b,β^	30.6 ± 2.1 ^a^	37.7 ± 1.8 ^a,b^	7.1 ± 1.0 ^a,b,β^ (0.20 ± 0.03)
**Group III ^5^**	2% RO, 1% FO and 0.1% CA (the CA diet ^6^)	0.174 ± 0.001 ^a^	30.6 ± 1.9 ^a^	37.2 ± 1.4 ^a^	6.6 ± 0.8 ^a^ (0.19 ± 0.02)
**Group IV ^5^**	2% RO, 1% FO, 0.1% CA and 0.35 mg Se as SeY in 1 kg BD (the CASeY diet ^6^)	0.174 ± 0.001 ^a^	30.3 ± 1.3 ^a^	36.8 ± 1.4 ^a^	6.5 ± 0.7 ^a^ (0.19 ± 0.02)
**Group V ^5^**	2% RO, 1% FO, 0.1% CA and 0.35 mg Se as Se (VI) in 1 kg BD (the CASeVI diet ^6^)	0.215 ± 0.001 ^b^	30.3 ± 2.1 ^a^	38.5 ± 2.7 ^b^	8.2 ± 0.7 ^b^ (0.23 ± 0.02)

Results are shown as means ± standard deviation (SD). Data with different superscripts (a, b) in columns, significantly (a, b *p* ≤ 0.05) differ from each other; trends were declared at 0.05  <  α, β *p* ≤ 0.10. ^1^ BW of sheep after the preliminary period. ^2^ BW of sheep fed for 35 days with the control diet (the RO diet), FO, CA, CASeY or CASeVI diets. ^3^ BWG  =  (BW_35days_ − BW_initial_); in parentheses: the average daily BWG of lambs (BWG/35). ^4^ During the preliminary period, animals were fed the basal diet (BD) enriched in 3% rapeseed oil (RO). ^5^ During the preliminary period, animals were fed the BD enriched in 1% fish oil (FO) and 2% RO; the iodine value of odorless FO were presented in our recent paper [41]. ^6^ The Se level in the RO, FO, and CA diets was 0.16 mg Se/1 kg BD; the Se level in the CASeY and CASeVI diets was 0.51 mg/1 kg BD.

**Table 2:** The MS/MS parameters for the experiment.

Analytes	Precursor Ion (m/z)	Product Ion (m/z)	Dwell Time (ms)	Q1 Voltage Potential (V)	CE Collision Energy (V)	Q3 Voltage Potential (V)
**SeMetSeCys ^1^**	183.90	167.00	100.0	−20.0	−10.0	−20.0
	183.90	94.95	100.0	−20.0	−25.0	−20.0
**SeCys2 ^2^**	336.80	247.90	100.0	−20.0	−15.0	−20.0
	336.80	88.15	100.0	−20.0	−25.0	−20.0
**SeMet** **^3^**	198.00	181.15	100.0	−25.0	−12.0	−30.0
	198.00	109.10	100.0	−24.0	−23.0	−18.0

^1^ SeMetSeCys-Se-methyl-Se-cysteine; ^2^ SeCys2-Se-cystine; ^3^ SeMet-Se-methionine.

**Table 3:** Quantification of total Se content and the selenium species in lamb tissue (results expressed per dry mass (dm)).

Experimental Diet		Total Se Content	Extracted SeCys2	Extracted SeMetSeCys	Extracted SeMet
		μg/kg dm	μg/kg dm	μg/kg dm	μg/kg dm
**Liver**	group I—RO diet	853.5 ± 192.2	51.2 ± 15.1	69.7 ± 16.6	49.9 ± 2.7
	group II—FO diet	700.1 ± 76.3	45.7 ± 16.3	73.3 ± 19.7	43.7 ± 5.4
	group III—CA diet	740.4 ± 113.4	55.2 ± 11.1	68.7 ± 11.1	49.7 ± 9.2
	group IV—CASeY diet	1381.4 ± 139.6	132.8 ± 12.3	166.2 ± 19.5	176.6 ± 14.8
	group V—CASeVI diet	1448.2 ± 179.2	44.7 ± 16.2	137.6 ± 12.6	103.1 ± 7.7
**Muscle** **MLD**	group I—RO diet	243.7 ± 31.3	52.0 ± 4.6	58.3 ± 10.1	64.5 ±7.9
	group II—FO diet	252.2 ± 25.0	60.7 ± 6.3	65.0 ± 15.2	60.1 ± 8.3
	group III—CA diet	257.1 ± 35.6	60.4 ± 2.3	65.5 ± 3.2	42.3 ± 20.9
	group IV—CASeY diet	352.5 ± 127.2	79.0 ± 4.1	156.1 ± 23.0	254.8 ± 11.3
	group V—CASeVI diet	317.7 ± 26.5	55.4 ± 3.6	50.9 ± 8.8	121.9 ± 16.9
**Heart**	group I—RO diet	767.1 ± 99.6	68.6 ± 4.6	62.2 ± 9.0	55.7 ± 4.9
	group II—FO diet	799.4 ± 121.7	68.9 ± 12.4	50.2 ± 10.6	43.2 ± 3.3
	group III—CA diet	871.2 ± 59.5	61.1 ± 15.2	47.9 ± 8.6	59.5 ± 3.0
	group IV—CASeY diet	1134.5 ± 142.4	92.1 ± 6.8	128.4 ± 6.5	89.0 ± 11.3
	group V—CASeVI diet	1138.5 ± 103.5	62.6 ± 3.5	60.3 ± 8.6	99.3 ± 12.4

Data are presented as mean (± SD) for measurements in three replicates for five animals undergoing the same treatment. dm–dry mass; MLD-longissimus dorsi muscle

## Supplementary Materials

The following are available online at https://www.mdpi.com/2076-2615/10/5/808/s1, Table S1: Chemical composition of the concentrate-hay diet with mineral-vitamin mixture in the basal diet (BD) and the control (RO), and experimental diets fed to lambs.
