# Supplementary material for: Determination of Selenium Species in Muscle, Heart, and Liver Tissues of Lambs Using Mass Spectrometry Methods
Source: Animals (Basel). 2020 May 7;10(5):808. doi: 10.3390/ani10050808 (PMC7277398; doi:10.3390/ani10050808)
Supplement: Supplementary file 1 [file animals-10-00808-s001.pdf]

Supplementary materials

Supplementary material associated with this article can be found, in the online version.

Table 1. Chemical composition of the concentrate-hay diet with mineral-vitamin mixture in the basal diet (BD) [1] and the control (RO), and experimental diets fed to lambs.

Specification	Meadow hay [1]	Concentrate[1]			Vitamins and mineral premix[2]
		Barley meal	Soybean meal	Wheat starch	
**Ingredient, g/kg BD**	360	165	360	90.0	20.0
**Dry matter (DM), %**	88.4	87.6	89.7	87.3	–
**In DM, %**					
**Crude protein**	9.50	9.94	41.8	0.90	–
**Crude fiber**	27.3	2.87	4.34	–	–
**Crude fat**	3.40	2.50	2.25	0.09	–
**Ash**	4.85	1.84	6.16	0.12	–
**Neutral detergent fiber**	59.2	18.0	18.8	–	–
**Acid detergent fiber**	32.1	4.61	6.44	–	–
**Acid detergent lignin**	4.47	1.14	1.49	–	–
**Gross energy, MJ/kg of DM**	17.1	16.3	17.8	16.7	–
**Chemical composition of the control and experimental diets**					
**Specification**	The control diet[3]		The experimental diets[4]		
**Dry matter, g/kg BD**	884		884		
**Crude protein, g/kg DM**	202		202		
**Crude fiber, g/kg DM**	119		119		
**Crude fat[5], g/kg BD**	21.7		21.7		
**Total crude fat[6], g/kg BD**	51.7		51.7		
**Ash, g/kg DM**	42.8		42.8		
**Neutral detergent fiber, g/kg DM**	311		311		
**Acid detergent fiber, g/kg DM**	146		146		
**Acid detergent lignin, g/kg DM**	23.3		23.3		
**Gross energy, MJ/kg DM[7]**	16.8		16.8		
**Total gross energy, MJ/kg DM[8]**	17.9		17.9		

- The concentrations of toxic elements in the basal diet (mg/kg): As, 1.39 ± 0.03; Cd, 0.068 ± 0.001; Sb, 0.0155 ± 0.0015; and Pb, 0.514 ± 0.003.
- [1]FA profiles in meadow hay, barley meal, soybean meal, and wheat starch were presented in the previous publication [1].
- [2]The concentrations of elements and vitamins in mineral-vitamin mixture were also presented in the previous publication [1].

- [3]The BD supplemented with 3% rapeseed oil (RO).
- [4]The FO diet: the BD supplemented with 2% RO and 1% odourless fish oil (FO); the experimental diets supplemented with antioxidants: the BD supplemented with 2% RO, 1% odourless FO and with the antioxidant(s) (i.e., carnosic acid (CA), selenized-yeast (SeY) or selenate (Se(VI))).
- [5]Crude fat originating from the BD (i.e., meadow hay and concentrate).
- [6]Total crude fat originating from BD and supplemented oils (i.e., RO without or with FO).
- [7]The gross energy of the BD (i.e., meadow hay and the concentrate) without supplementation with RO, FO, CA and Se (as SeY or Se(VI)).
- [8]Total gross energy of the BD supplemented with RO without or with FO and antioxidant(s) (i.e., CA, SeY, or Se(VI)).

RO comprised the following main fatty acids (μg/g RO): C14:0 56, C16:0 13091, c9C16:1 33, C18:0 5490, c9C18:1 85859, c12C18:1 786, c9c12C18:2 282394, c9c12c15C18:3 74, C20:0 194, c11C20:1 108, C22:0 430 and c15C24:1 61.

FO included the following main fatty acids (μg/g): C12:0 82, C14:0 12345, c9C14:1 215, C15:0 477, C16:056947, c7C16:1 318, c9C16:1 420,ΣC16:2 15586, C17:0 493, c9C17:1 193, C18:0 9452, c6C18:1 188, c7C18:1 842, c9C18:1 290592, c12C18:1 15834, c14C18:1 159, c9c12C18:2 114512, c9c12c15C18:3 20968, c11C20:1 24206, c7c9c12c15C18:4 473, c11c14C20:2 2270, c8c11c14C20:3 258, c5c8c11c14C20:4 304, c8c11c14c17C20:4 607, C22:0 139, c13C22:1 11036, c11C22:1 1704, c5c8c11c14c17C20:5 6792, c13c16C22:2 95, c7c10c13c16C22:4 144, c15C24:1 397, c7c10c13c16c19C22:5 1560 and c4c7c10c13c16c19C22:6 26570.

The vitamin and mineral mixture was purchased from POLFAMIX OK (Grodzisk Mazowiecki, Poland); 1 kg of vitamin and mineral mixture comprised: 285 g calcium, 16 g phosphorus, 56 g sodium, 42 mg cobalt as carbonate, 10 mg iodine as iodate, 1 g iron as sulphate, 6mg Se as selenite, 0.5 g copper as sulphate, 5.8 g manganese as sulphate, 7.5 g zinc as sulphate; vitamins: A (500,000 IU/kg), D3 (125,000 IU/kg), and E as α-tocopherol (25,000 IU/kg).

The selenized yeast (SeY; Se-Saccharomyces cerevisiae) was donated by Sel-Plex (Alltech In., USA). About 83% of the Se content of selenized yeast (SeY) represents Se in the form of Se-methionine (Se-Met) incorporated into the proteins of S. cerevisiae.

Reference

[1] D. Jaworska, M. Czauderna, W. Przybylski, A.J. Rozbicka-Wieczorek, Sensory quality and chemical composition of meat from lambs fed diets enriched with fish and rapeseed oils, carnosic acid and seleno-compounds, Meat Sci. 119 (2016) 185–192. doi:10.1016/j.meatsci.2016.05.003.
